# Evaluation of anti-biofilm activity of acidic amino acids and synergy with ciprofloxacin on *Staphylococcus aureus* biofilms

**DOI:** 10.1038/s41598-020-66082-x

**Published:** 2020-06-02

**Authors:** Annsar A. Warraich, Afzal R. Mohammed, Yvonne Perrie, Majad Hussain, Hazel Gibson, Ayesha Rahman

**Affiliations:** 1 Aston Pharmacy School, Aston University, Birmingham, B4 7ET UK; 2 University of Strathclyde, Glasgow, G1 1XQ Scotland; 3 Quest Healthcare Ltd, Birmingham, UK; 4 University of Wolverhampton, WV1 1LY Wolverhampton, UK

**Keywords:** Antibiotics, Biofilms

## Abstract

Acidic amino acids, aspartic acid (Asp) and glutamic acid (Glu) can enhance the solubility of many poorly soluble drugs including ciprofloxacin (Cip). One of the mechanisms of resistance within a biofilm is retardation of drug diffusion due to poor penetration across the matrix. To overcome this challenge, this work set to investigate novel counter ion approach with acidic amino acids, which we hypothesised will disrupt the biofilm matrix as well as simultaneously improve drug effectiveness. The anti-biofilm activity of D-Asp and D-Glu was studied on *Staphylococcus aureus* biofilms. Synergistic effect of combining D-amino acids with Cip was also investigated as a strategy to overcome anti-microbial resistance in these biofilms. Interestingly at equimolar combinations, D-Asp and D-Glu were able to significantly disperse (at 20 mM and 40 mM) established biofilms and inhibit (at 10 mM, 20 mM and 40 mM) new biofilm formation in the absence of an antibiotic. Moreover, our study confirmed L-amino acids also exhibit anti-biofilm activity. The synergistic effect of acidic amino acids with Cip was observed at lower concentration ranges (<40 mM amino acids and <90.54 µM, respectively), which resulted in 96.89% (inhibition) and 97.60% (dispersal) reduction in CFU with exposure to 40 mM amino acids. Confocal imaging indicated that the amino acids disrupt the honeycomb-like extracellular DNA (eDNA) meshwork whilst also preventing its formation.

## Introduction

Biofilms are communities of sessile microorganisms (bacteria or fungi) living in a self-produced matrix (matrix of extracellular polymeric substances) which aids the survival of these microorganisms^1,2^. The formation of biofilm can be divided into stages. Firstly planktonic bacteria attach to a surface reversibly^1,3^. At this stage the bacteria are still susceptible to antibiotics. Hence prophylaxis in alloplastic surgery proves very useful at this stage since the antibiotics are able to act on the planktonic forms which become reversibly attached^3,4^. In the absence of a challenge during the next stage, the bacteria become irreversibly attached through self-adhesion structures such as pili, multiply and form colonies. Furthermore, extracellular polymeric substances, which form the matrix are secreted and deposited around the colonies^3,4^. The matrix is a key structural component of the biofilm community and consists of water, polysaccharides, proteins, lipids and DNA^3,4^. Hydrogen bonding between water molecules and hydrophilic polysaccharides holds water within the biofilms whilst polymers such as glycopeptides, lipids and lipopolysaccharides aid in scaffolding and holding the biofilm together^3,4^. As the biofilm grows thicker, it becomes mature; a process which is coordinated through quorum sensing and other signalling pathways^3,4^. Finally, the sessile bacteria in the biofilm are able to become free and spread to other surfaces to form new biofilms^1,3,4^.

Biofilm forming bacteria are commonly associated with chronic infections, which are infections that persist despite antibiotic therapy and the body’s immune response^4^. This is because the therapy and immune response are able to overcome planktonic bacteria, but are unable to penetrate the biofilm defences. After a while, when the individual seems to be cured, the sessile communities in biofilms give rise to planktonic forms, which in turn leads to a relapse of the disease state^1^. Since the bacteria in biofilms have evolved complex survival strategies, biofilms act as a persistent base which poses constant pathogenicity to the body through continuous release of virulence factors and planktonic forms^4^. Hence a chronic state of infection is maintained due to the inability of the immune system or drugs to eradicate the biofilm.

Biofilm formation on abiotic surfaces can result in complications with implants as well as catheters^2^. A good example which highlights the danger associated with biofilms is a biliary stent in which the growth of *Escherichia coli* (*E.coli*) biofilm lead to repeated incidents of sepsis. In this case, antibiotic therapy was of no avail and the second episode of sepsis proved fatal. DNA typing revealed that the same *E.coli* clonal type was present in the biofilm as the one responsible for sepsis^4^. On the other hand, biofilms are also implicated on biotic surfaces such as acute and chronic wounds. In such cases, along with the disease state of infection, biofilms also hinder wound healing^5^.

The problem is exacerbated by the fact that resistance to antibiotics can be up to 1000 times higher for bacteria in biofilms and the underlying mechanisms of resistance are likely to be a combination of conventional resistance mechanisms found in planktonic phenotypes as well as those specific to biofilm phenotypes^1,2,6^. Such specific mechanisms include retardation of antibiotic diffusion by the biofilm matrix as well as slowed growth of bacteria due to decreased metabolic rate in biofilms^4,6^. The latter leads to resistance because most antibiotics have their effect on rapidly dividing cells^1^.

There is an urgent need therefore for strategies which can successfully and safely prevent as well as those which can treat infections where biofilms are implicated. The strategies studied can be divided into four major categories; prevention of biofilm formation, weakening of the biofilm, disruption or dispersal of the biofilm and killing of bacteria particularly the subpopulation which persists^1^.

One strategy utilises D-amino acids to inhibit and disperse biofilms. Their role was seen after D-leucine, D-methionine, D-tryptophan and D-tyrosine, isolated prior to disassembly of *Bacillus subtilis* biofilms, were found to prevent new biofilm formation as well as cause its dispersal. It was found that D-amino acids caused cells in the biofilm to release amyloid fibres which are involved in linking cells in the biofilm together. YqxM is a protein required in the formation and anchoring of the Tas A amyloid fibres (amyloid fibres which in their composition contain Tas A protein) to the cell wall^7^. These amyloid fibres give structural integrity to the biofilm. Strains with mutations in this protein were able to form biofilms in the presence of D-amino acids, indicating that the anti-biofilm activity of these amino acids was dependent on the strain with the wild type YqxM protein^8^.

Since this finding, D-amino acids have also proved to be effective as strategies for the prevention and dispersal of *Staphylococcus epidermidis, Staphylococcus aureus* (*S. aureus*) *and Pseudomonas aeruginosa* biofilms^9,10^. Studies have demonstrated their effectiveness in combination with antimicrobials in a bid to overcome the antimicrobial resistance of biofilms^9^. It is generally claimed that L-amino acids have no effect and literature attributing anti-biofilm activity to these isoforms is limited^8,11,12^.

*S. aureus* was used in this study as it is one of the most clinically prominent bacteria and is frequently the causative agent in both acute and chronic infections. Along with this, the WHO has listed *S. aureus* as a high priority pathogen to help focus research and development against AMR^13^. Its infections can range from relatively mild skin infections to life-threatening infections such as endocarditis and pneumonia^14^. Strains such as methicillin-resistant *S. aureus* (MRSA), have emerged which are resistance to multiple antibiotics and infections caused by MRSA result in poorer clinical outcomes for the patient^15^. Furthermore, other D-amino acids have been shown to prevent its biofilm formation as well as cause its dispersal. *S. aureus* NCTC 8325, a strain with a well-defined genotype, was used for this investigation.

Acidic amino acids are able to increase the solubility of Ciprofloxacin (Cip)^16^. This can probably be attributed to the acidic side chain which both aspartic acid and glutamic acid possess. Given the pK_a_ values of these side chains, they are likely to exist as charged anionic species at and around neutral pH. Thus it was hypothesised that their physiochemical properties and the resulting solubility enhancement, along with their potential anti-biofilm activity, can be utilised as a dual approach to decrease antimicrobial resistance within bacterial biofilms, through amino acid-drug synergy.

## Materials and Methods

### Materials

*S. aureus* strain NCTC 8325 was obtained from Public Health England. Tryptone soya broth (TSB), tryptone soya agar (TSA), Ciprofloxacin 98%, D(-)-aspartic acid 99 + %, D(-)-glutamic acid 99 + % were all obtained from Fisher Scientific whereas L-aspartic acid 98 + % and L-glutamic acid 99% were obtained from Sigma Aldrich. Crystal violet (CV) was obtained from Sigma Aldrich. Phosphate buffered saline (PBS) tablets and LIVE/DEAD^TM^ BacLight^TM^ Bacterial Viability Kit were obtained from Thermofisher Scientific.

### General methods

The biofilm formation, quantification and dispersal assays were adapted from published protocols^9,17,18^. For inoculum preparation, 10 ml TSB was inoculated with a colony of *S. aureus* and incubated overnight at 37 °C in an aerobic orbital shaker at ~50 cycles/min. The optical density (OD) was measured using a spectrophotometer at a wavelength of 600 nm, and the culture was diluted with TSB to adjust the OD to 0.1. Minimum lethal concentration (MLC) of Cip against planktonic forms of *S. aureus* was previously determined and found to be 3.75 ×10^−6^ g/ml.

### Biofilm dispersal assay

The biofilms were grown for appropriate number of hours (6, 12, 24, 48 and 72 h) before addition of the amino acids. To do this, 500 µl of the diluted culture was added to each well of the 24 well plate. The plates were then incubated in a static incubator for 37 °C.

To examine the dispersal effects of amino acids, 40 mM solutions of aspartic acid (Asp), glutamic acid (Glu) and aspartic acid combined with glutamic acid (AA) were prepared in the broth followed by filter sterilisation using 0.22 µm syringe filters. Broth was used for serial dilution to attain working concentrations of 40, 20, 10, 5, 2.5 mM for the amino acids from the stock solution. After 72 h of incubation, the wells were washed once with PBS. 500 µl of media with various concentrations of amino acids were added to the wells. Negative control was TSB without any amino acids; both for the 72 h biofilm growth and later washed for and TSB added for overnight treatment experiment. The plates were then incubated overnight at 37 °C, washed and quantified by crystal violet staining. Cip and Cip in combination with amino acids was assessed in a similar way.

### Biofilm inhibition assay

To determine the inhibitory effects of amino acids, Cip and Cip combined with AA, the biofilms were allowed to grow whilst being exposed to these test substances from the start of the experiment. For this the culture was mixed with the D-amino acids before it was incubated for 6, 12, 24, 48 and 72 h. Briefly, after achieving appropriate culture (0.1 OD) and test substance concentration within each well, the plates were left in a 37 °C incubator for the desired time before the biofilm density was quantified. Again for negative control biofilms, TSB lacking any test substance was used.

### Crystal violet staining

The media was removed from the plates through gentle shaking into a beaker and followed by gentle dabbing of the plates on a bed of paper towels to remove excess media. Each well was then washed once with 500 µl PBS followed by staining with 500 µl of 0.1% CV (w/v) for 30 min. The wells were then washed three times by submerging the plates in a tub of distilled water and by gently dabbing in a bed of paper towels to remove any excess stain. The plates were then left overnight or for a few hours (until dry) before proceeding.

Once dry 625 µl of 30% (v/v) acetic acid was added to each well to dissolve the CV. Plates were left to incubate for 15 min after which the solubilised crystal violet was transferred into a new plate and the biofilm was quantified by determining the OD using a plate reader at the wavelength of 570 nm.

### Colony forming units

To determine the colony forming units (CFU), excess media was removed from the wells and the wells were washed with PBS once as described earlier. Next 500 µl of TSB was added to each well and the biofilm was resuspended in the media by scraping and re-pipetting. This detached the biofilm from the well surface whilst also breaking up any free floating aggregates of the biofilm in the media. After vortexing the suspension, the Miles and Misra method was used to determine the CFU/ml.

### Rate of cell attachment

Rate of cell attachment to a glass surface was determined through live imaging obtained using ZEISS Celldiscoverer 7. Images were obtained every 5 min over 2.5 h. Since the focus of lens was set at the surface of the glass, the clearly visible cells were taken to be those which had attached to the surface. ZEISS Celldiscoverer 7 software was used to quantify the number of cells which had attached.

### Fluorescence and confocal imaging of biofilms

Glass coverslips were sterilised with 70% (v/v) ethanol and placed in wells of 6 well plates. Biofilms were grown on coverslips as detailed under biofilm inhibition assay and washed with 0.85% (v/v) sodium chloride whilst dabbing on a bed of paper towel to maximise drainage. Dyes SYTO 9 and propidium iodide (PI) from the LIVE/DEAD BacLight Bacterial Viability Kit were used to stain the cells. Staining solution was made in deionised water at a concentration of 3 µl/ml of each dye from a stock of 3.34 mM SYTO9 and 20 mM PI. The plates were then incubated for 90 min at 37 °C with 500 µl of the staining solution in each well. The wells were then rinsed with RNA free water, and the plates left upside down to drain for 10–15 min. The coverslip were then mounted onto slides using BacLight mounting oil. The biofilm samples were then analysed using confocal microscopy using CLSM TCS SP5 II system (Leica Microsystems GmbH, UK) using a x63 and a x100 oil immersion lens. An argon laser was used to excite SYTO 9 fluorophores and a helium-neon laser was used to excite PI fluorophores at a wavelength of 488 nm and 543 nm respectively. The emission analyser filters were set between the range of 493 nm to 530 nm for SYTO 9 and 625 nm to 676 nm for PI. For fluorescence imaging, biofilms were stained in a similar way within the wells of a 24 well plate and analysed on the same day using Leica Widefield Fluorescence Microscope (Leica Microsystems GmbH, UK). ImageJ (https://imagej.nih.gov/ij/index.html) was used to quantify the percentage of eDNA present within AA and Cip treated biofilms compared to control.

### Statistical analysis

One-way ANOVA was followed by a post-hoc t-test Bonferroni correction to determine where the significant difference lay within the data set. P value <0.05 was taken to be significant. Data presented is the mean of at least three replicates (from independent bacterial biofilms) unless otherwise stated. Biological replicates (from independent bacterial cultures) were conducted for experiments with D-AAs and L-AAs and they gave reproducible biofilm density. Thereafter, technical replicates were done whilst including these controls in the experiments, to ensure reproducibility.

## Results

### Biofilm inhibition and dispersal using D-Asp and D-Glu

**Figure 1:**
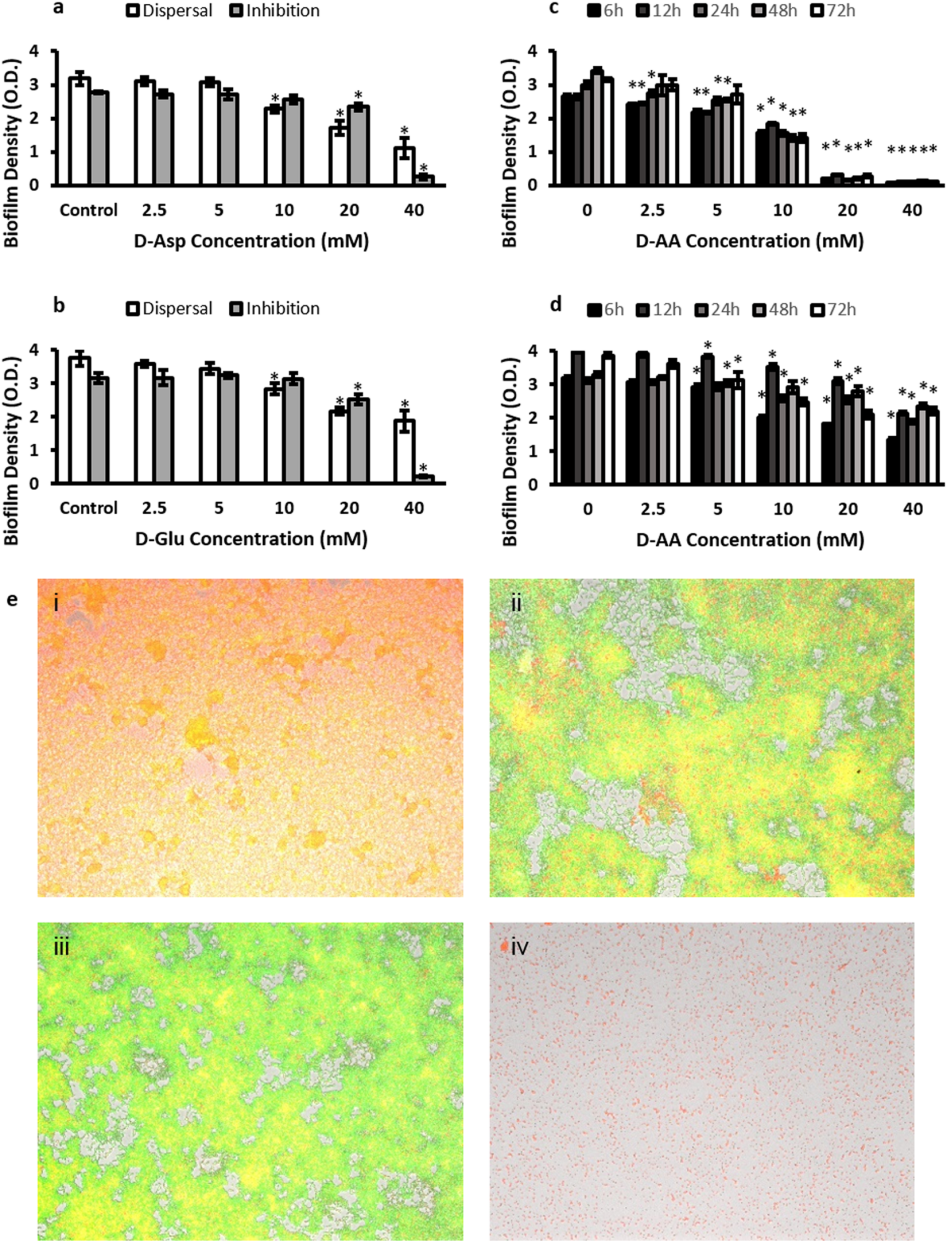
(**a**) Inhibition and dispersal of *S. aureus* biofilm using 2.5, 5, 10, 20 and 40 mM D-Asp. **(b)** Inhibition and dispersal of *S. aureus* biofilm using 2.5, 5, 10, 20 and 40 mM D-Glu. **(c)** Inhibition of *S. aureus* biofilm using equimolar concentrations of D-AA. **(d)** Dispersal of *S. aureus* biofilm using equimolar concentrations of D-AA. **(a-d)** One-way ANOVA showed an overall significant difference within the data and was followed by a post-hoc t-test with Bonferroni correction to see which concentration significantly (p < 0.0083 was taken as significant; indicated by *) significantly inhibited or dispersed biofilms; n = 3, ±S.D. **(e)** Fluoresence imaging showing 72 h biofilm formation. **(i)** Control **(ii)** inhibited with 40 mM D-Asp **(iii)** inhibited with 40 mM D-Glu **(iv)** inhibited with equimolar concentrations of D-Asp and D-Glu (40 mM of each); D-Asp = D-Aspartic acid; D-Glu = D-Glutamic acid; D-AA = D-Asp and D-Glu.

The concentration of amino acids used were 2.5 mM, 5 mM, 10 mM, 20 mM and 40 mM. Upper limit was capped at 40 mM considering the solubility limitations of these amino acids in water. Figures 1a,b show the effect on biofilm density of D-Asp and D-Glu whilst they were used separately as inhibiting and dispersing agents. Concentration dependent anti-biofilm activity (Fig. 1a–d) was observed with each time point (Fig. 1c,d) whether amino acids were used in isolation or in combination. However, greater anti-biofilm activity (Fig. 1c,d) was observed by combining D-Asp and D-Glu (D-AA). When combined, 2.5 mM, 5 mM, 10 mM, 20 mM and 40 mM concentrations of AA represent pH values of 7.15, 6.86, 6.51, 5.76, 4.59 and 4.01 respectively. Figure 1a–d also show that D-AA were effective in both preventing (inhibition) the formation of new biofilms, as well as the breakdown (dispersal) of already formed biofilms. Significant inhibition (p < 0.001) was achieved from the lowest concentration (2.5 mM) of D-AA used whereas minimum of 5 mM D-AA concentration was required to obtain statistically significant (p < 0.05) dispersal. The maximum inhibition and dispersal effect was obtained with the higher concentrations of D-AAs used i.e. 20 mM and 40 mM. The D-AA were better at inhibition than dispersal. Anti-biofilm activity was investigated on biofilms grown for 6, 12, 24, 48 and 72 h. The amino acids were similar in effectiveness at all the time points. Therefore, considering that bacteria are most resistant once the biofilm has matured, the 72 h time point was considered appropriate for conducting further work^19^. For biofilms grown for 72 h, at 40 mM 37.55% of biofilm was dispersed whereas 96.89% was inhibited from forming (Fig. 1c,d).

Fluorescence images in Fig. 1e confirm that both D-Aspartic acid and D-Glutamic acid have anti-biofilm activities and that combining both amino acids is more efficacious. High levels of extracellular DNA (eDNA) was evident in the untreated biofilm (Fig. 1e i) compared to those treated (Fig. 1e ii,iii,iv). Use of amino acids leads to patchy (Fig. 1e ii,iii) and incomplete (Fig. 1e iv) biofilm formation. Equimolar combination of both amino acids was therefore chosen for further investigations. The work presented here was obtained using combination of both aspartic acid and glutamic acid such that if 40 mM D-AA is stated, it means that 40 mM of D-Asp and 40 mM of D-Glu was used together.

### L-AA also inhibit and disperse biofilms

**Figure 2:**
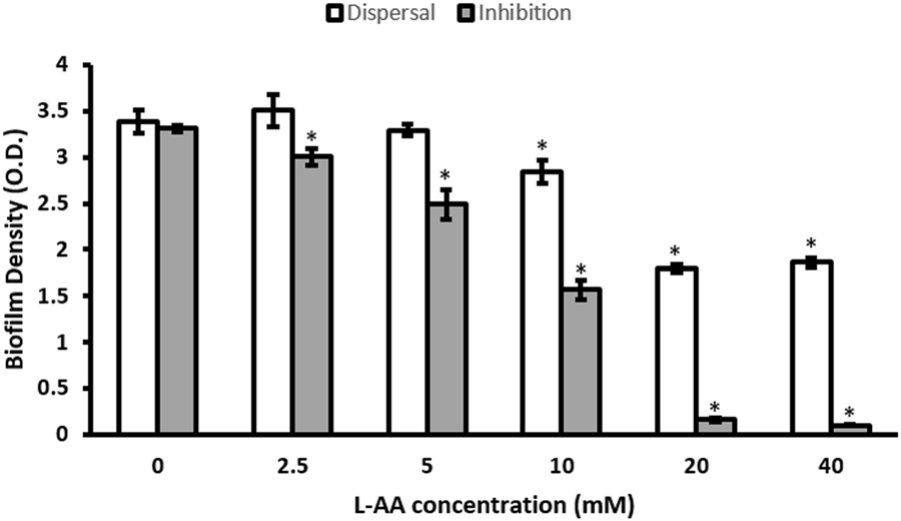
Dispersal and Inhibition of *S. aureus* biofilm using L-AA (equimolar combination of L-Asp and L-Glu) was dependent on the concentration of amino acids used. One-way ANOVA showed an overall significant difference within the data and was followed by a post-hoc t-test with Bonferroni correction to see which concentration significantly (p < 0.0083 was taken as significant; indicated by *) significantly inhibited and dispersed biofilms; n = 3, ±S.D; L-Asp = L-Aspartic acid; L-Glu = L-Glutamic acid; L-AA = L-Asp and L-Glu.

It was important to investigate whether the anti-biofilm activity of D-AA is restricted to the isomeric form. For this, combination of L-Asp and L-Glu (L-AA) were investigated for their anti-biofilm properties, whilst using the same concentrations as D-AA. L-AA showed a similar, concentration dependent dispersal and inhibition profile (Fig. 2) to D- isoforms whilst inhibiting and dispersing biofilms.

### Synergistic effect of D-AA and Cip on biofilm inhibition and dispersal

**Figure 3:**
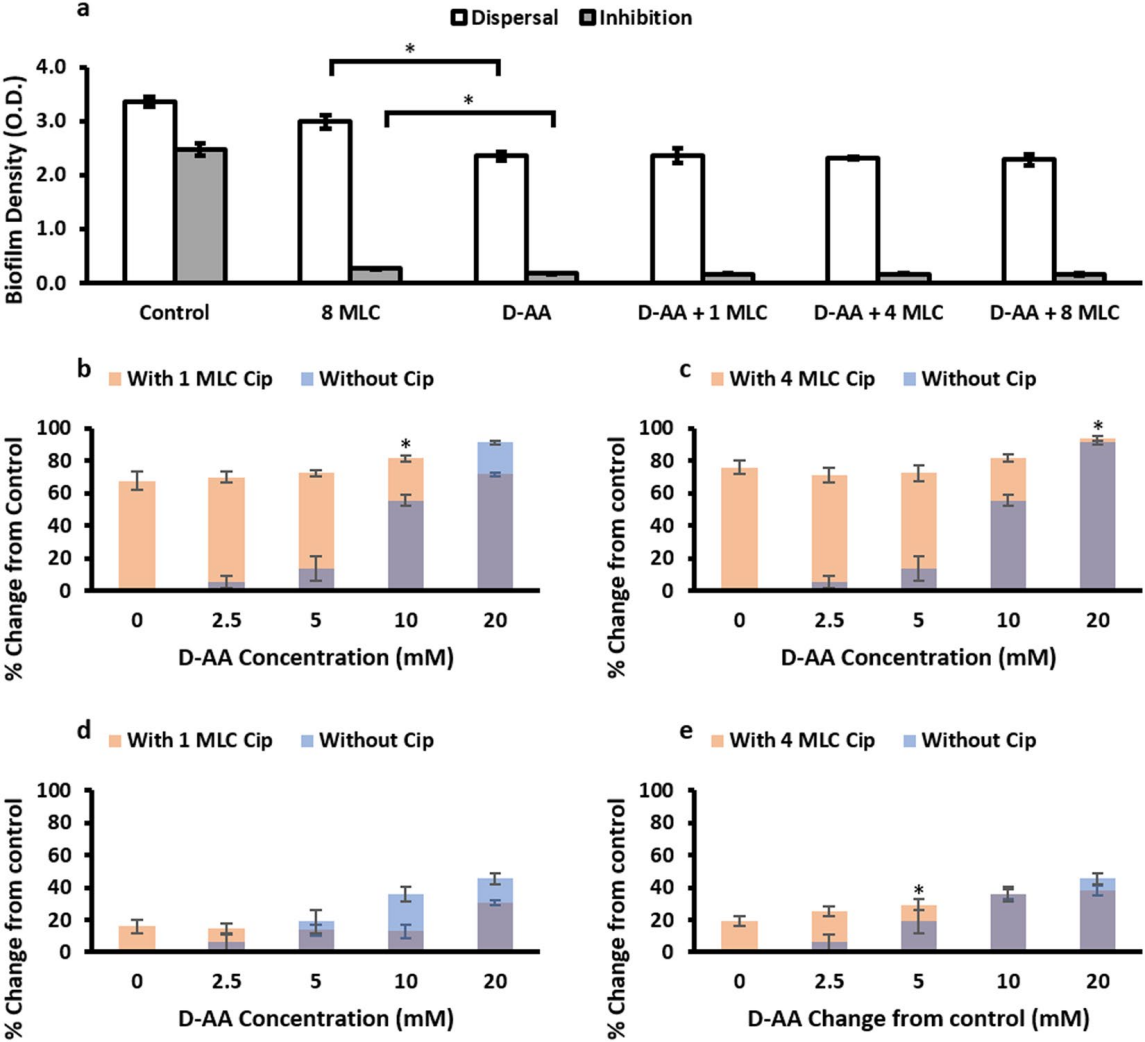
(**a**) Efficacy of 8 MLC Cip, 40 mM D-AA and 40 mM D-AA combined with 1 MLC, 4 MLC and 8 MLC Cip on biofilm density whilst inhibiting and dispersing *S. aureus* biofilms. Two tailed T-test revealed that D-AA have a significantly (indicated by *) greater anti-biofilm activity (inhibition p < 0.001, dispersal p < 0.0001) as compared to 8x MLC of Ciprofloxacin. One-way ANOVA showed no significant synergistic effect (p > 0.05) on biofilm density between D-AA and when D-AA and Cip are used in combination. **a-e)** Synergistic effect of using combination of 1 MLC **(b,d)** or 4 MLC **(c,e)** Cip with 2.5, 5, 10 and 20 mM D-AA on biofilm inhibition **(b,c)** and dispersal **(d,e)**. One-way ANOVA showed an overall significant difference for amino D-AA + Cip data **(b-e)**. This was followed by a post-hoc t-test with Bonferroni correction to see which concentration of D-AA + Cip had significantly higher (p < 0.01) anti-biofilm activity as compared Cip on its own. Next one-tail t test was done to see whether this combination of D-AA + Cip had significantly (p < 0.05) improved anti-biofilm activity as compared to the corresponding D-AA on its own. **(b)** 10 mM D-AA + 1 MLC Cip was significantly (*) able to inhibit greater biofilm formation compared to both Cip and D-AA on their own (p < 0.001 and p < 0.00001 respectively). **(c)** 20 mM D-AA + 4 MLC Cip was significantly (*) able to inhibit greater biofilm formation compared to both Cip and D-AA on their own (p = 0.00014 and p = 0.014 respectively). **(d)** No combination was significantly able to disperse greater biofilm compared to both Cip and D-AA on their own **(e)** 5 mM D-AA + 4 MLC Cip gave significant (*) rise in biofilm dispersal activity compared to both Cip and D-AA on their own (p = 0.0045 and p = 0.018 respectively). **(a-e)** D-AA = 40 mM D-Aspartic acid and 40 mM D-Glutamic acid; x MLC = x times minimum lethal concentration; Cip = ciprofloxacin; n = 3, ±S.D.

Although, Cip on its own also has anti-biofilm activity, potential synergistic effect of using Cip with amino acids was investigated. Anti-biofilm activity of Cip and D-AA alone was compared to combined D-AA (2.5–40 mM) + Cip (1 MLC, 4 MLC and 8 MLC; number represents x times MLC) at various concentration combinations (Fig. 3a–e). 40 mM D-AA showed significantly greater biofilm inhibiting (p < 0.001) and dispersing (p < 0.0001) activity as compared to 8 MLC Cip. Whilst 40 mM D-AA + Cip (8 MLC, 4 MLC or 1 MLC) maintained the anti-biofilm activity of the amino acid, there was no synergistic effect observed of these combinations on biofilm density (Fig. 3a). However, at specific combinations with lower concentrations of D-AA, significant synergistic (p < 0.05, when compared to both the corresponding D-AA concentration as well as the Cip concentration) effect was observed (Fig. 3b–e); 10 mM D-AA + 1 MLC Cip and 20 mM D-AA + 4 MLC Cip showed significant synergy in biofilm inhibition whilst 5 mM D-AA + 4 MLC Cip showed significant synergy in biofilm dispersal.

### Effect on colony forming units

**Figure 4:**
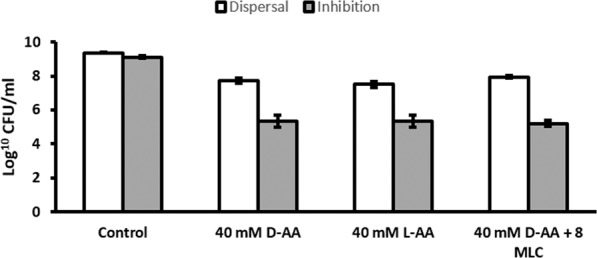
Effect of 40 mM D-AA, 40 mM L-AA and 40 mM D-AA + 8 MLC on CFU after biofilm inhibition and dispersal. ≥99.85% (inhibition) and ≥91.89% (dispersal) reduction in CFU from control was observed for D-AA, L-AA and D-AA + 8 MLC; D-AA = 40 mM D-Aspartic acid and 40 mM D-Glutamic acid; x MLC = x times minimum lethal concentration of ciprofloxacin; n = 3, ±S.D.

Since biofilms are composed of bacterial cells and extracellular matrix, it was necessary to distinguish how the anti-biofilm activity of amino acids was distributed between these two components. Measuring biofilm density is a quantitative method that does not distinguish between matrix and cells. To determine the effect of amino acids and ciprofloxacin on the viability of cells residing in *S. aureus* biofilms, viability assays were carried out by estimating the CFU/mL. Figure 4 compares the log_10_ CFU/ml of untreated biofilms with biofilms treated with 40 mM D-AA, 40 mM L-AA, and 40 mM D-AA + 8 MLC Cip. Experiments for dispersal showed greater than 1 log reduction (≥91.89%) decrease in log_10_ CFU/ml for biofilms dispersed with 40 mM D-AA and 40 mM L-AA, as compared to untreated biofilms. Whereas for inhibition using the same treatments, there was greater than 2 log reduction (≥99.85%) in the number of viable bacterial cells attached to the surface.

### D-AA treatment prevents eDNA meshwork formation

**Figure 5:**
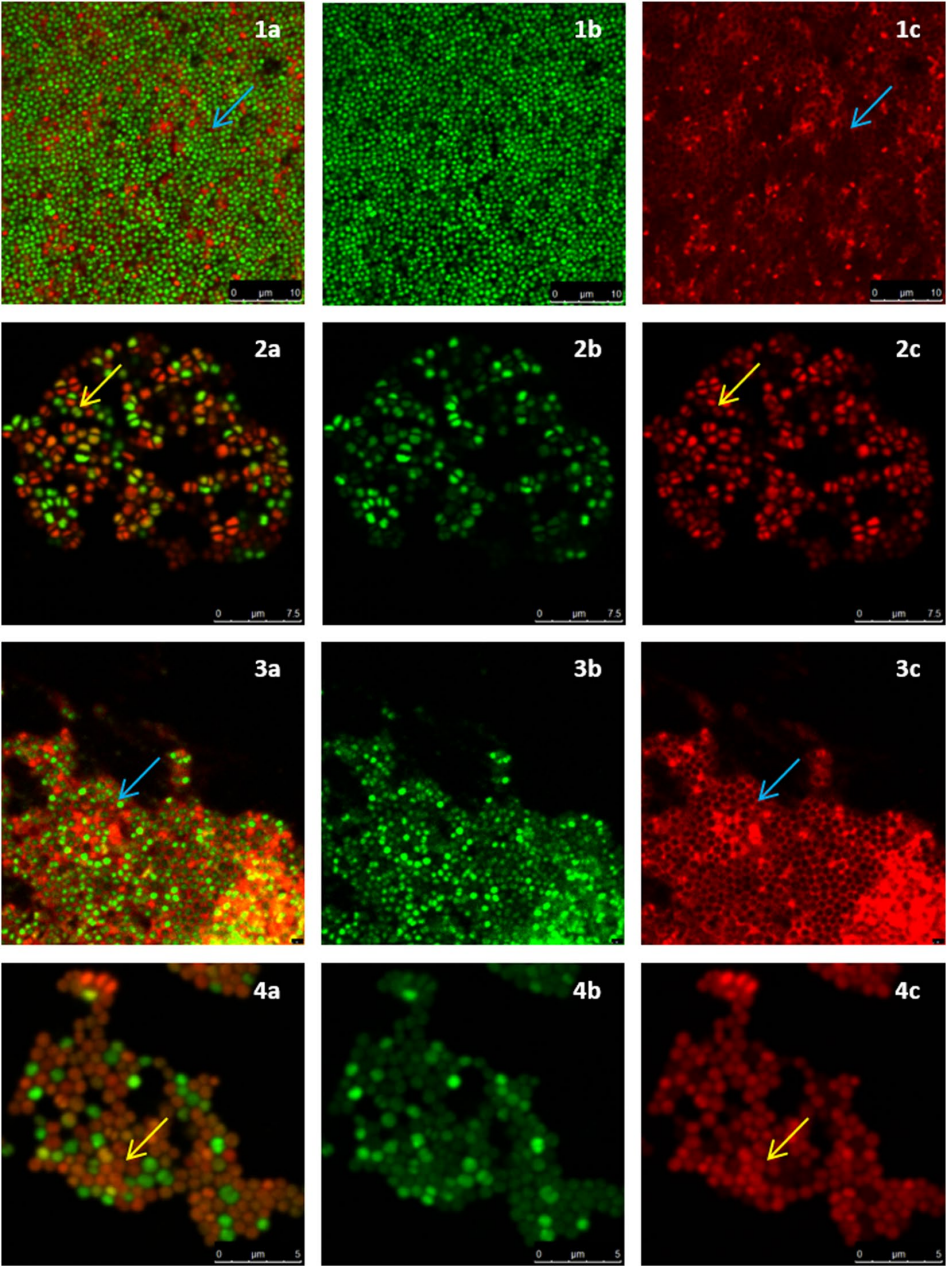
Confocal images of inhibited *S. aureus* biofilms. **(a)** combined channel; **(b)** SYTO 9 channel; **(c)** Promidium Iodide channel; **(1)** control biofilm; **(2)** inhibited with 40 mM D-AA (40 mM D-Asp and 40 mM D-Glu); **(3)** treated with Cip; **(4)** treated with 40 mM D-AA and 8 MLC Cip; blue arrow) presence of intercellular eDNA; yellow arrow) lack of intercellular eDNA. **(1)** Untreated *S. aureus* biofilms are reinforced by an organised honeycomb like meshwork made of interconnected intercellular eDNA. eDNA forms a filamentous mesh like structure within the biofilm and surrounds all cells. **(2)** Biofilms inhibited with D-AA show a complete lack of eDNA. **(3)** Biofilms inhibited by Cip express a lower population of *S. aureus* cells. A dense eDNA meshwork provides structure to these persisting cells. **(4)** Biofilm treated with a combination of D-AA and 8 MLC Cip lack in eDNA meshwork structure; D-AA = 40 mM D-Aspartic acid and 40 mM D-Glutamic acid; x MLC = x times minimum lethal concentration; Cip = ciprofloxacin.

**Figure 6:**
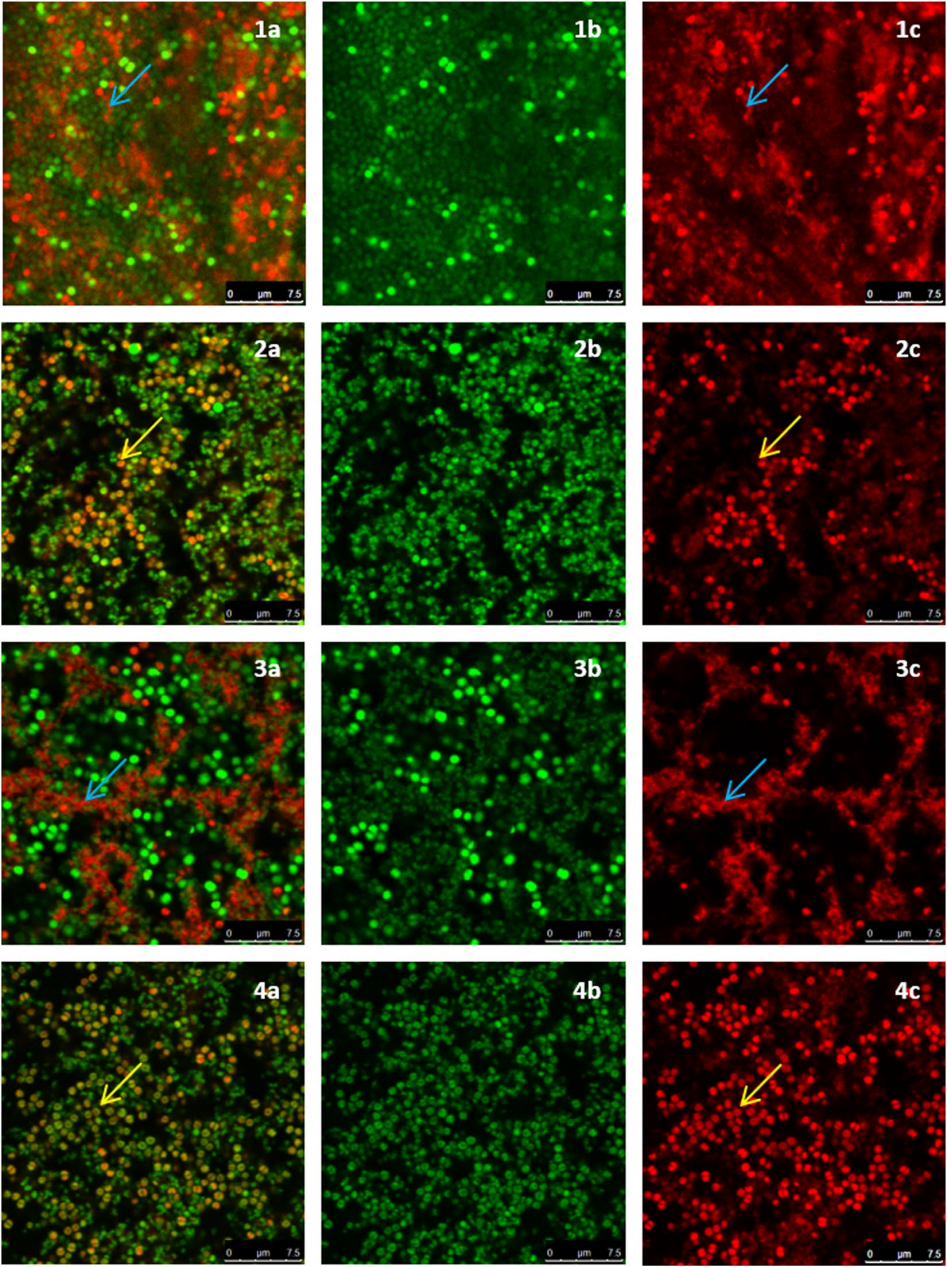
Confocal images of dispersed *S. aureus* biofilms. **(a)** combined channel; **(b)** SYTO 9 channel; **(c)** Promidium Iodide channel; **(1)** control biofilm; **(2)** dispersed with 40 mM D-AA (40 mM D-Asp and 40 mM D-Glu); **(3)** dispersed with Cip; **(4)** dispersed with 40 mM D-AA and 8 MLC Cip; blue arrow) presence of intercellular eDNA; yellow arrow) lack of intercellular eDNA. **(1)** Untreated *S. aureus* biofilms are reinforced by an organised honeycomb like meshwork made of interconnected intercellular eDNA. eDNA forms a filamentous mesh like structure within the biofilm and surrounds all cells. **(2)** Biofilms dispersed with D-AA lack majority of the eDNA. The pan-biofilm eDNA network is lost, any remaining eDNA is likely to be that entrapped between other matrix substances. **(3)** Presence of eDNA networks provides structure to persisting cells in biofilms treated with Cip on its own. **(4)** Biofilm treated with a combination of D-AA and 8 MLC Cip lack in eDNA meshwork structure and have minimal eDNA; eDNA = extracellular DNA; eDNA = extracellular DNA; D-AA = 40 mM D-Aspartic acid and 40 mM D-Glutamic acid; x MLC = x times minimum lethal concentration; Cip = ciprofloxacin.

For confocal imaging, biofilms were grown on glass coverslips. After the treatment experiments, biofilms were stained with the dyes SYTO 9 and PI which stain nucleic acids in live and dead cells respectively. When both dyes are used in conjunction, the SYTO 9 channel generally represents the nucleic acid which is present within intact membranes and the PI channel enables the visualisation of nucleic acids outside cells (eDNA) or cells with a compromised cell membrane. The images taken through confocal microscopy are presented in Figs. 5, 6 and Supplementary Fig. 1. Images of inhibited (Fig. 5) and dispersed (Fig. 6) biofilms from both, the SYTO 9 channel and the PI channel along with their overlay are presented. In this way the structure of eDNA can be more easily visualised whilst also being able to see how it spatially relates to both live and dead cells.

Untreated *S. aureus* biofilm covers the whole field of view and it has the highest cell density. In comparison, cells within all inhibited and dispersed biofilms have a patchy or clustered distribution. *S. aureus* cells in the control biofilms are interconnected and held in position by an organised distribution of eDNA throughout (Fig. 5.1 and 6.1). This meshwork of eDNA is filamentous in nature and almost seems to represent a honeycomb like structure. Furthermore, there is a uniformity in the thickness of eDNA between cells within the biofilms, raising the question as to whether the intercellular distance within the biofilm is precisely controlled and not without purpose.

From visual analysis of the confocal images it is clear that both inhibited and dispersed biofilms exposed to D-AA, whether in isolation (Fig. 5.2 and 6.2) or in combination with Cip (Fig. 5.4 and 6.4), not only lack this organised structure of eDNA, but lack eDNA altogether. This was quantified using ImageJ (supplementary Table 3), revealing treated biofilms to possess 98.04% ± 0.67 (AA) and 95.76% ± 1.66 (Cip) less eDNA compared to control when inhibited or dispersed respectively. Considering the clear lack of eDNA within inhibited biofilms, any eDNA present within biofilms dispersed by these agents is most probably a result of being entrapped within the complex matrix. Like cells within biofilms treated with D-AA and D-AA + Cip, *S. aureus* cells within biofilms treated with Cip on its own are also distributed in clusters. However, Cip-only treated biofilms do not lack eDNA (Fig. 5.3, 6.3 and supplementary Table 3). Moreover, the eDNA which is present in these biofilms seems to be more densely structured as compared to that of untreated biofilms. Dispersing with Cip-only also reveals an interesting feature; the eDNA seems to be present as patches or in boundaries, with number of viable cells decreasing as we move away from these boundaries (Fig. 6.3 and Supplementary Fig. 1).

### D-AA reduce the rate of cell attachment

**Figure 7:**
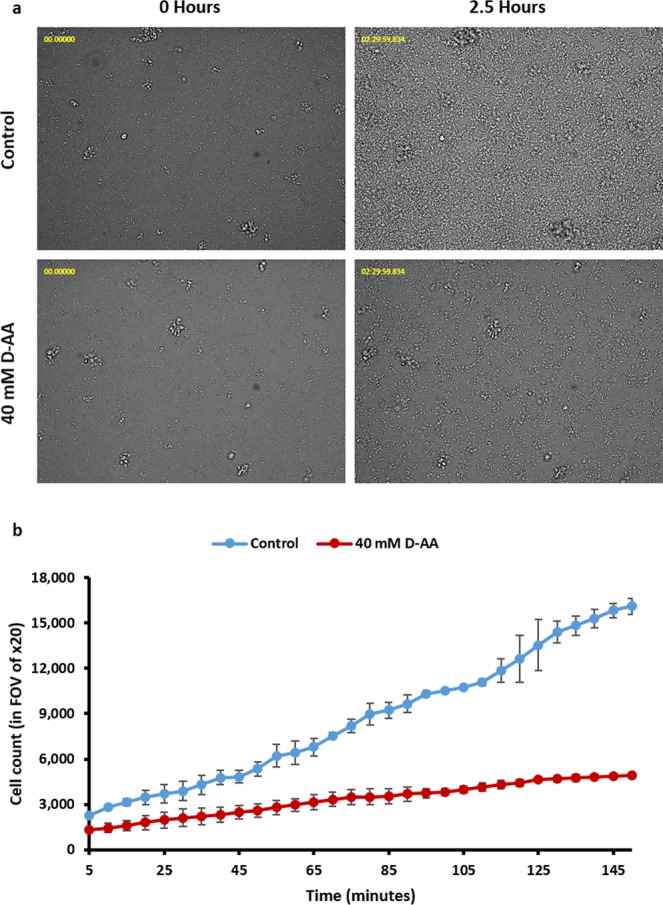
**(a)** 40 mM D-AA significantly reduce the number of cells attached to the plate surface over 2.5 hours of incubation. **(b)** The rate of *S. aureus* attachment is also slower in cells treated with 40 mM D-AA as compared to control; D-AA = 40 Mm D-Aspartic acid and D-Glutamic acid. FOV = field of view; n = 2, ±S.D.

Since attachment of cells to a surface is the key initial stage of biofilm formation, it was appropriate to investigate the effect of D-AA on cell attachment. Figure 7 shows that exposing *S. aureus* cells to 40 mM D-AA reduces the rate of cell attachment compared to untreated cells. Throughout the attachment assay, a gradual increase in the percentage reduction in cell attachment was observed, starting from 43.45% at 5 min to 69.50% at 150 min.

## Discussion

Aspartic acid and glutamic acid are known to enhance the solubility of Cip^16^. Considering that one of the mechanisms of antimicrobial resistance in biofilms is the hindrance of drug diffusion through their matrix, it was decided to utilise these solubility enhancers of Cip in order to overcome this mechanism of antimicrobial resistance in *S. aureus* biofilms. Along with this, since amino acids themselves are known to possess anti-biofilm activity, whether D-Asp and D-Glu confer anti-biofilm activity was also investigated, whilst also studying the nature of this activity.

Although the effectiveness of D-isoforms of amino acids as anti-biofilm agents is somewhat under debate^8,11,12^, much attention has been given to them over the past decade and their role in inhibiting and dispersing biofilms. On the other hand, most publications agree on a lack of anti-biofilm activity of L-amino acids, and some even attribute pro-biofilm activity to them^20–22^. Literature claiming that L-amino acids have anti-biofilm activity is rare^11,12^. It was therefore appropriate to choose D-isoforms as solubility enhancers for Cip. A hypothesis was drawn that any potential anti-biofilm activity specific to D-isoforms would supplement the above proposed biofilm perfusion enhancing mechanism and may further enhance the efficacy of Cip.

Due to age dependent variation in cells and matrix of a biofilm, Vidakovic *et al*. (2018), hypothesized that susceptibility to the anti-microbial agent (phage) may vary depending on age of the biofilm. They found that younger biofilms (<48 h) were more susceptible to phage activity than older (>60 h) ones^23^. Similarly, Yang *et al*. (2015) found that younger biofilms (24 h) were more susceptible to inhibition by D-Asp than biofilms grown for 48 h and 72 h^12^. Data presented here shows that whilst inhibiting, generally the anti-biofilm activity of D-AAs is independent of age, since in most concentrations, the percentage reduction in biofilm formation is similar regardless of how long the biofilm was incubated for (supplementary Fig. 2). On the other hand, dispersing already formed biofilm becomes less effective as the biofilm ages from 6 h to 48 h. At 72 h, however the efficacy of amino acids again rises (supplementary Fig. 3), probably due to endogenous dispersal mechanisms also being involved at this point.

It seems Tong *et al*. (2014) were the first to attribute anti-biofilm activity to L-amino acids, shortly followed by Yang *et al*. (2015)^12^. Whilst Yang *et al*. (2015) found that D and L-Asp were able to inhibit and disperse *S. aureus* biofilms, Tong *et al*. (2014) only reported inhibition of *Streptococcus mutants* biofilms^11,12^. However, the latter found inhibition with both D and L-isoforms of both of the acidic amino acids. In this paper we show that either isoform of both Asp and Glu are able inhibit and disperse *S. aureus* NCTC 8325 biofilms (Figs. 1–6). To our knowledge, this is a previously unreported biofilm forming strain to be tested with these amino acids.

Contrary to our findings with *S. aureus*, L-Glu has been reported to enhance biofilm formation of *Azotobacter chroococcum (A. chroococcum)*. The concentration (40 mM) we present to be most potent in anti-biofilm activity, Velmourougane *et al*. (2017) show, possess the highest pro biofilm activity^22^. However their results in another paper seem to suggest that eDNA may be low if at all present in *A. chroococcum* biofilms^24^. Furthermore, it seems that the importance of eDNA in forming biofilms and their role in stability after biofilm formation varies from species to species^25^. Absence of eDNA is probably unlikely, as even without any specific DNA releasing mechanisms, eDNA is a natural product of dead cells. It is more likely therefore, that eDNA does not play a pivotal role in the formation of *A. chroococcum* biofilms, rendering the anti-eDNA mechanism of L-Glu presented later on in this paper unchallenged. Hence, with no disruption to biofilm structure, the pro-biofilm effects L-Glu become more pronounced.

The ability of the dyes SYTO 9 and PI to distinguish between live and dead cells can be attributed firstly, to the inability of PI to enter live cells possessing intact membranes (except in exceptional cases), whereas SYTO 9 is able to enter both live and dead cells^26,27^. Secondly, PI has higher affinity for, and is able to, displace SYTO 9 from nucleic acids^27^; despite the presence of SYTO 9 within dead cells or within extracellular matrix, SYTO 9 is unable to compete against PI for nucleic acids. Hence, DNA within both dead cells and the extracellular matrix (eDNA) is detected through the fluorescence of PI bounded to it. This is made feasible by the fact that the fluorescence of both dyes is substantially lower when unbound; fluorescence of both dyes is enhanced greatly when the dyes are attached to nucleic acids. As a result, despite the presence of SYTO 9, it is not detected in the presence of PI. This allowed confident localisation of eDNA through confocal imaging (Figs. 5 and 6). These dyes have been previously used by Okshevsky *et al*. to visualise eDNA^28^. It seems that due to specific conditions PI may also enter live cells. Kirchhoff *et al*. (2017) showed that PI can enter live cells if there are changes to membrane potential, resulting in yellow/orange cells as opposed to the expected green or red^26^. It is likely that the orange cells seen in Fig. 5.2 and 6.2 are due to some uptake of PI which binds to DNA within the cells^26^. Since the rest of the DNA within the cells has SYTO 9 bounded to it, fluorescence of both red and green makes cells appear orange in the overlay. This uptake of PI through intact membranes may be due to a change of membrane potential or an increase in the permeability of PI through other mechanism caused by amino acids^26,29^.

With amino acid exposure, one cause for the reduction in biofilm CFU/ml and in the rate of cell attachment (Figs. 4 and 7) is likely due to reduction in planktonic cell viability (supplementary Fig. 4). However another important cause suggested by fluorescence and confocal findings is lack of eDNA (Figs. 1e, 5 and 6). Recently, Sugimoto *et al*. (2018) showed that eDNA is the most common component of *S. aureus* biofilms suggesting it to be a prime target for anti-biofilm therapeutics^30^. This paper proposes that a way to target this vital structural matrix component is acidic amino acids; the biofilms inhibited and dispersed with D-AA at 40 mM concentration were unable to form (Fig. 5.2) or lost (Fig. 6.2) their eDNA honeycomb meshwork, respectively. Since eDNA is important in initial bacterial attachment to a surface and also for further biofilm growth, including cell aggregation, the lack of eDNA or its inability to form a structural framework means that the biofilm is neither able to build a strong foundation nor is able to mature, ultimately stunting its growth^31,32^. Furthermore, with the continuous role of aggregating cells and providing stability to the already formed biofilms, lack of eDNA leads to the breakdown of already formed biofilms, at least partially. Thus the low number of viable cells in inhibited biofilms can be attributed to the inability of the cells to attach and aggregate. Whereas in dispersed biofilms, this would be due to destabilisation of the eDNA mediated intercellular bonding mechanism; the cells are released from the biofilm due to the lack of eDNA which would otherwise hold them together.

Whilst the cells within a *S. aureus* biofilm are interconnected through cell wall structures, matrix components also play a major role in the stabilisation of the biofilm^33^. The major components of the extracellular matrix in *S. aureus* biofilms are the polysaccharide intercellular adhesin (PIA) and as aforementioned, extracellular DNA (eDNA). The mechanisms involved in the release of eDNA from *S. aureus* cells is not yet clear. However it seems to be highly dependent on autolysis which has been attributed to many systems including those leading to a reduction in cell wall integrity^34,35^. As discussed later, findings reported in this paper highlight that eDNA release may also be derived by externally influenced lysis. Nevertheless, this eDNA has been described as an electrostatic net that anchors cells together^32^. However, for eDNA to serve its purpose in the matrix, *S. aureus* seems to employ certain moonlighting proteins i.e. enolase and GAPDH (glyceraldehyde-3-phosphate dehydrogenase) which are attached to the cell surface^36^. Although these proteins are cytoplasmic in origin, they serve a second function in the matrix acting as a bridge between the cells and the eDNA. The removal of these proteins by increasing the pH causes the release of eDNA from the *S. aureus* biofilms; showing that the role of eDNA in the structural stability of the biofilm is dependent on these cell surface proteins^32^. These proteins are proposed as an ideal bridge because they are thought to be positively charged, and hence are able to interact with and connect the negatively charged cells on one side with the negatively charged eDNA on the other^32^.

**Figure 8:**
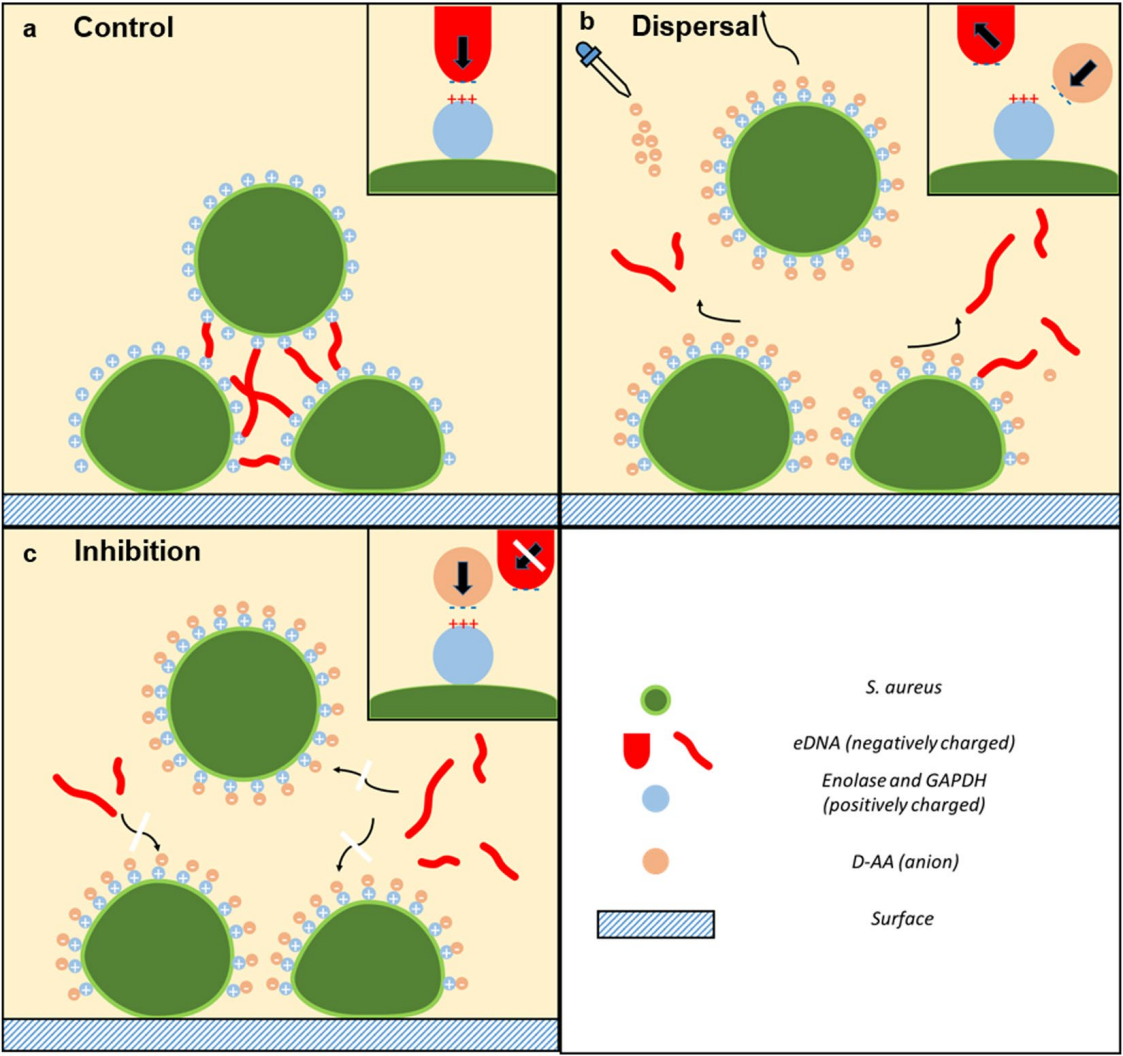
Mechanism of biofilm inhibition and dispersal using D-AA. D-AA exhibit anti-eDNA activity. **(a)** Normal conditions: eDNA forms intercellular bridges by interacting with surface bound positively charged proteins (enolase and GAPDH) via acid-base interactions. **(b)** Dispersal: addition of anionic D-AA displaces eDNA from the surface bound positively charged proteins. eDNA is released and the intercellular bridges are broken. **(c)** Inhibition: the present D-AA interact with surface bound positively charged proteins preventing eDNA from associating with these proteins; intercellular bridges are unable to form; eDNA = extracellular DNA; D-AA = D-Aspartic acid and D-Glutamic acid; GAPDH = glyceraldehyde-3-phosphate dehydrogenase.

It seems that eDNA in a healthy biofilm surrounds all cells individually (Fig. 5.1 and 6.1), helping them aggregate and provides structural stability and integrity to the biofilm. Das *et al*. (2011) suggest that bacterial aggregation mediated by eDNA is due to acid-base interactions^37^. We propose that acidic amino acids prevent the formation of eDNA meshwork, and if already present, break it down by interfering with these acid-base interactions. The mechanism that may be employed by acidic amino acids is depicted in Fig. 8. Negatively charged eDNA helps aggregate cells by interacting with positively charged *S. aureus* surface proteins (enolase and GAPDH) on multiple cells; thus acting as an intercellular electrostatic bridge (Fig. 8a). The importance of these proteins in intercellular bridging is highlighted by their presence, at varying concentrations, along the entire surface of bacteria^38–44^. Since at the pH value of 4.01, the ionised acidic amino acids are negatively charged, they are able to interact with these positively charged proteins. Resulting ion-pair formation, neutralises the positive charge on enolase and GAPDH, rendering eDNA unable to interact with these proteins. Therefore, during inhibition intercellular bridges do not form, neither are cells able to aggregate (Fig. 8c). Dispersal with amino acids utilises a similar mechanism. Anionic amino acids displace enolase and GAPDH associated eDNA. Release of eDNA causes a release of cells from the biofilm since they are now unable to aggregate with each other (Fig. 8b). Greater effectiveness of D-AA in combination as compared to their use individually can be explained through the presence of a greater number of anions in combination. More anions present greater obstruction for eDNA-protein interaction, resulting in combined D-AA exhibiting greater anti-eDNA activity. Support for the role of charge in the underlying mechanism is provided by the observation that neutralising the acidic amino acids using NaOH halts anti-biofilm activity (supplementary Fig. 5) of the amino acids.

In dispersed biofilms, it is likely that other matrix components are still present which would explain why there is a greater percentage reduction in viable cell number (Fig. 4 and supplementary Table 1) as compared to total biofilm density (Fig. 1 and supplementary Table 1). This is because with the release of eDNA, the cells are unable to stay aggregated and are released from the biofilm. However, other matrix components remain unaffected and constitute to the remaining biofilm density (Fig. 1a,b,d,e and supplementary Fig. 3) observed after dispersal experiments. This also gives reason as to why D-AA seem appear more efficacious at reducing biofilm density whilst inhibiting than whilst dispersing. Conversely, cell number and eDNA are not the only effected components in inhibited biofilms. Clearly, other matrix components are also unable to become established and form an unavoidable part of the percentage reduction in biofilm density (supplementary Table 2). This further highlights the role of eDNA in not only the aggregation of cells but also the establishment of other matrix components.

Confocal images show that use of treatment with Cip on its own leads to biofilms with seemingly denser eDNA structures (Fig. 5.3 and 6.3). This is a visual reminder as to why biofilms are a major cause of chronic infections. Whilst an antibiotic may get rid of majority of the bacterial load from the body, persister cells remain unharmed within biofilms. Biofilms are then able to re-establish once treatment is stopped and ultimately, the infection recurs. Interestingly, treating already formed biofilms with Cip alone reveals eDNA localised in a seemingly well-fortified boundaries (Fig. 6.3 and supplementary Fig. 1). Here we hypothesis two potential implications of these findings. Firstly, these boundaries suggest that within a biofilm eDNA structures have weak and strong points. Cells away from these boundaries are released upon treatment giving rise to voids within treated biofilms. Since these boundaries seem circular in morphology, they may be boundaries to microcolonies within the biofilm. Previous studies have shown eDNA to be highly concentrated around *Pseudomonas aeruginosa* microcolonies^45,46^. A second possibility is that upon threat, cells which are to persist within the biofilm are able to further fortify and make dense the eDNA structures surrounding them. This is made feasible due to the excess of eDNA present as a result of antibiotic triggered cell death. In either case, it is appropriate to speculate that these strong points within eDNA meshwork are likely responsible for cell persistence and antimicrobial resistance.

Despite the ability of acidic amino acids to enhance the solubility of Cip^16^, no synergistic effect of combining 40Mm D-AA and Cip was found in biofilm density or on cell viability. The reasons may be different for inhibition and dispersal. When inhibiting biofilms, 40Mm amino acids are alone able to inhibit 96.89% of the biofilm from forming. This leaves minimal room for improvement for any amounts of Cip which may be added in biofilms in combinations with the amino acids. Similarly, with a reduction of 97.60% CFU in biofilms dispersed with D-AAs, there are not many viable cells within the biofilm left for Cip to act on. In either case, any effect of Cip has probably gone unnoticed. Therefore it seemed likely that a synergistic effect may be more pronounced at lower concentrations of D-AAs. Further experiments were carried out which confirmed that at lower but specific concentrations, a synergistic effect on biofilm density is achievable by combining Cip with D-AA (Fig. 3b,c,e).

## Conclusion

Acidic amino acids are able to disperse mature biofilms and inhibit new *S. aureus* biofilm formation. This coincides with a lack of eDNA present in treated biofilms, suggesting that eDNA is a target for the observed D-AA anti-biofilm activity. Hence, an anti-eDNA mechanism is proposed; D-AA modulate the acid-base interactions which are essential for anchoring eDNA to cells. The findings further confirm that eDNA is critical to *S. aureus* biofilms, both in formation stages as well as once established. Targeting matrix components not only helps fight biofilms on its own, but may also be used to increase the efficacy of antimicrobial agents. Furthermore, the role of antibiotics in increasing anti-microbial resistance is reiterated, whilst suggesting that the mechanisms of acquiring anti-microbial resistance is not limited to cells but may extend to the matrix. The broader applicability of this mechanism in clinical strains of *S. aureus* such as the methicillin resistant *Staphylococcus aureus* strains will be studied to further consolidate the proposed mechanism of action, thereby widening the use of amino acids as potential anti-biofilm agents. Therefore, to fight anti-microbial resistance, drugs that target cells alone should be used in conjunction with ways to disrupt the matrix or inhibit its formation. The findings from this work can lead to applications where amino acids act as anti-biofilm agents to coat catheters, implants and in wound dressings.

## Supplementary information

Supplementary information.

## Data Availability

The datasets generated during and/or analysed during the current study are available from the corresponding author on reasonable request.
