# Supplementary material for: Evaluation of anti-biofilm activity of acidic amino acids and synergy with ciprofloxacin on Staphylococcus aureus biofilms
Source: Sci Rep. 2020 Jun 2;10:9021. doi: 10.1038/s41598-020-66082-x (PMC7265346; doi:10.1038/s41598-020-66082-x)
Supplement: Supplementary file 1 — Supplementary information. [file 41598_2020_66082_MOESM1_ESM.pdf]

Supplementary Information for:

Evaluation of anti-biofilm activity of acidic amino acids and synergy with ciprofloxacin on Staphylococcus aureus biofilms

Annsar A Warraich1, 4, Afzal Mohammed1, Yvonne Perrie2, Majad Hussain3, Hazel Gibson4, Ayesha Rahman*4

[1]Aston Pharmacy School, Aston University, Birmingham, B4 7ET, UK; [2]University of Strathclyde, Glasgow, G1 1XQ; [3]Quest Healthcare Ltd, Birmingham, UK; [4]University of Wolverhampton, WV1 1LY, UK

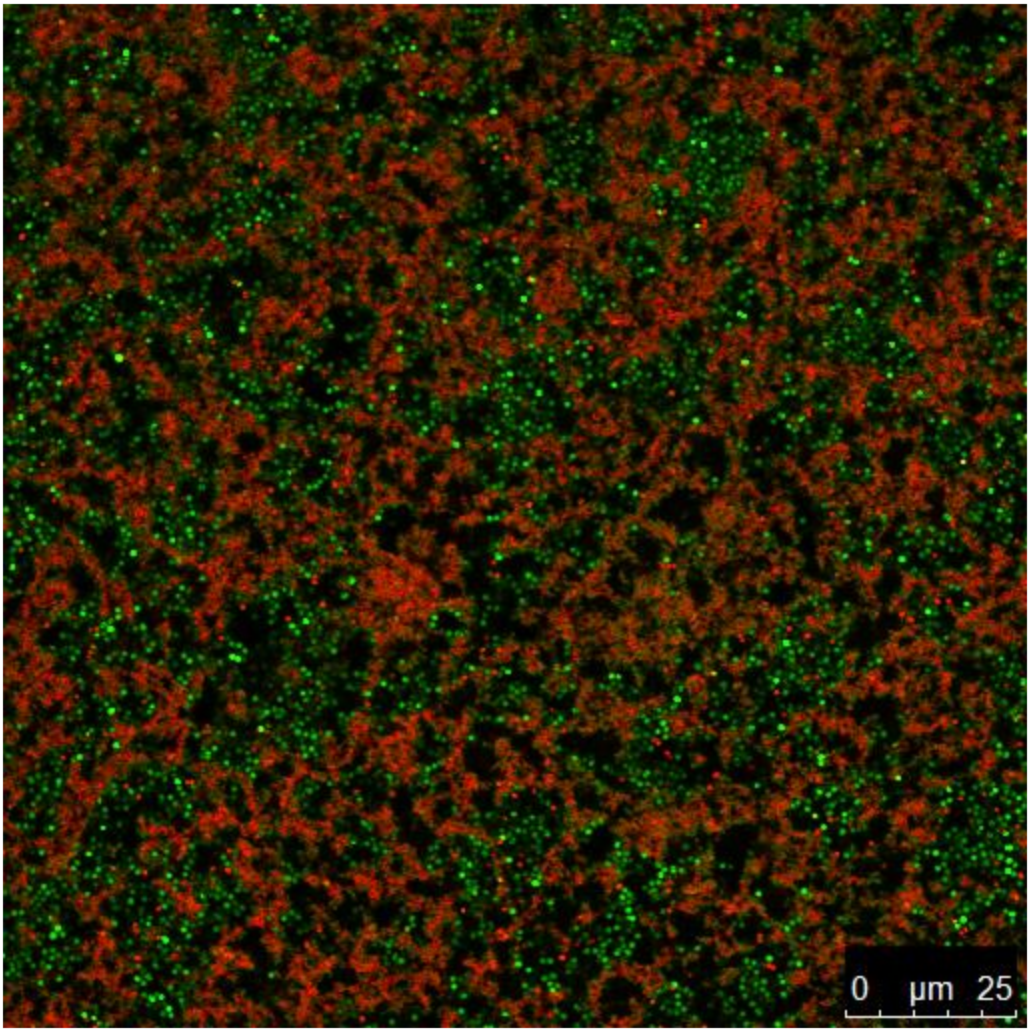

Supplementary Figure 1: Confocal Image of biofilm dispersed with Cip alone. eDNA is present as seemingly well fortified boundaries, enclosing persisting *S. aureus cells*.

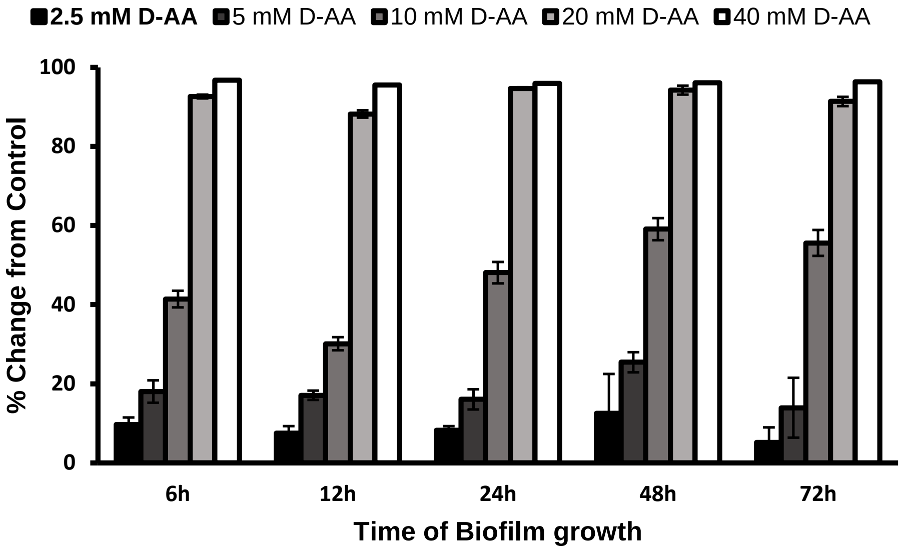

Supplementary Figure 2: Percentage change from control upon biofilm inhibition; n=3.

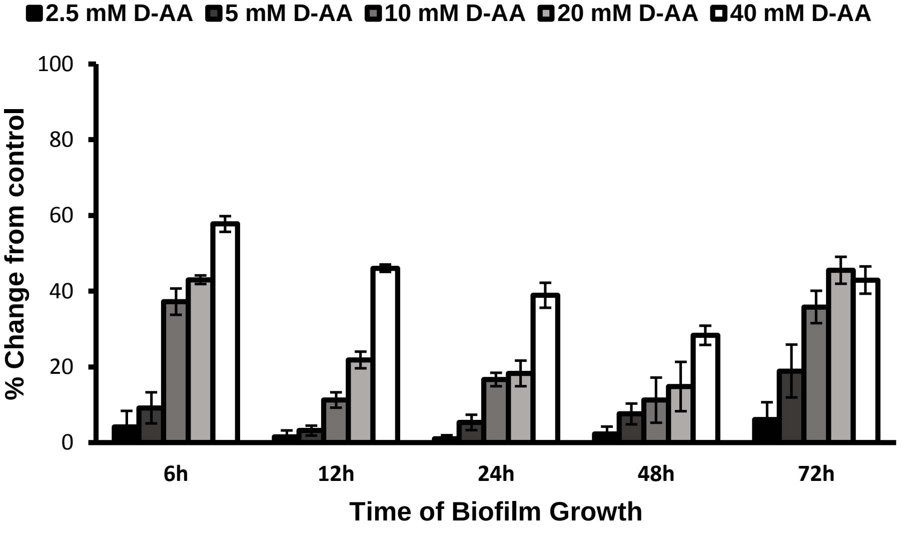

Supplementary Figure 3: Percentage change from control upon biofilm dispersal; n=3.

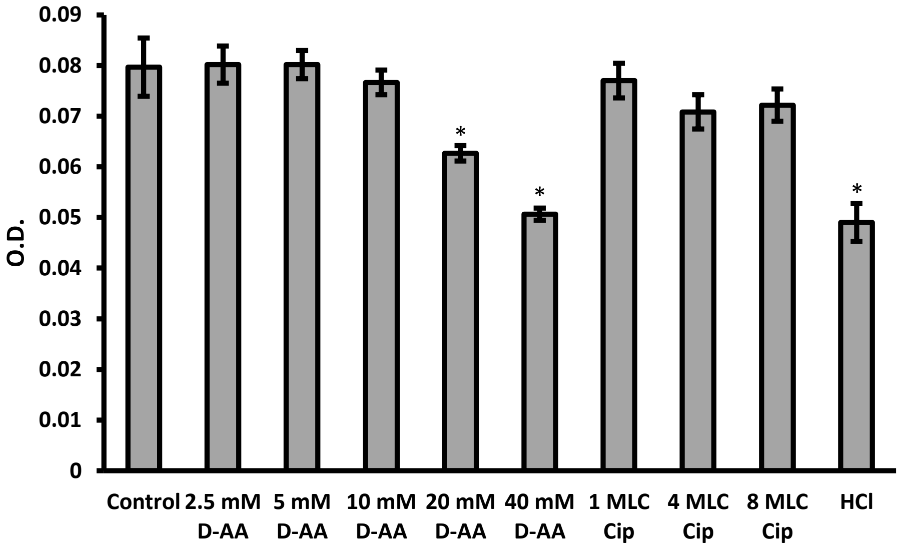

Supplementary Figure 4: Effect of D-AA at concentrations of 2.5, 5, 10, 20 and 40mM, and Cip at concentrations of 1 MLC, 4 MLC and 8 MLC on the Optical density of planktonic S. aureus growth after 30 minutes of exposure. One-way ANOVA showed an overall significant difference within the data and was followed by a post-hoc t-test with Bonferroni correction to see which concentration significantly ($p<0.005$ was taken as significant; indicated by *) killed or effected planktonic growth of S. aureus compared to the control. Significant effect on planktonic growth of S. aureus was observed with 20mM and 40mM D-AA; n=6.

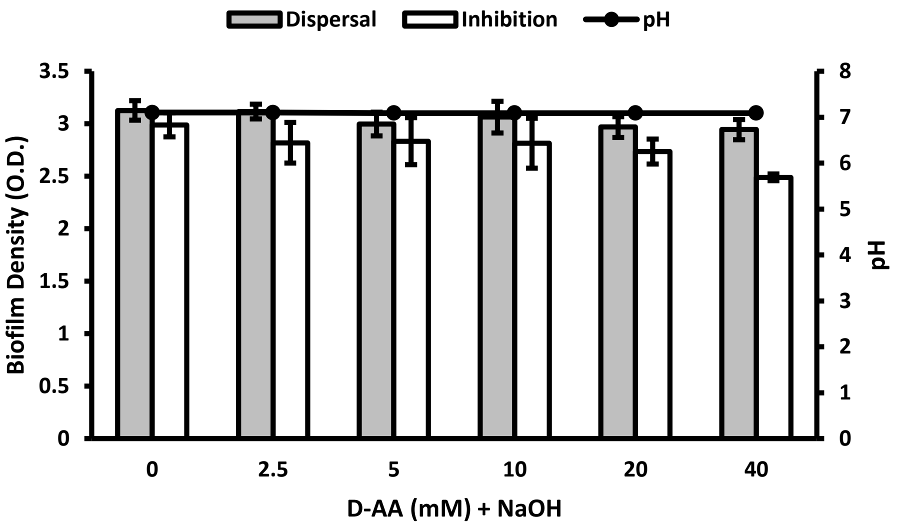

Supplementary Figure 5: Effect of NaOH on the anti-biofilm activity of D-AA. Neutralising acidic amino acids using NaOH resulted in the anti-biofilm activity of these agents to be halted; n=4.

Supplementary Table 1: Comparison of percent change in biofilm density with percent change in viable cells for dispersed biofilms. There is a greater reduction in number of viable cells as compared to the reduction in total biomass. This pattern is more prominent in dispersed biofilms than inhibited.

Dispersing agent	Percent reduction in biofilm density	Percent reduction in cell viability
**40mM D-AA**	30.16	97.59
**40mM L-AA**	44.92	98.55
**40mM D-AA + 8 MLC**	32.00	96.09

Supplementary Table 2: Comparison of percent change in biofilm density with percent change in viable cells for inhibited biofilms. There is a greater reduction in number of viable cells as compared to the reduction in total biomass. This pattern is more prominent in dispersed biofilms than inhibited.

Inhibiting agent	Percent reduction in biofilm density	Percent reduction in cell viability
**40mM D-AA**	92.78	99.98
**40mM L-AA**	97.01	99.98
**40mM D-AA + 8 MLC**	93.58	99.99

Supplementary Table 3: Percentage of eDNA present in AA and Cip treated biofilms as compared to treated biofilms was quantified using ImageJ. Table also presents percentage reduction in eDNA compared to control.

	**Inhibiton**		**Dispersal**	
	% of eDNA compared to control	% reduction in eDNA compared to control	% of eDNA compared to control	% reduction in eDNA compared to control
**Control**	100	0	100	0
**AA**	1.96 ± 0.67	98.04 ± 0.67	4.24 ± 1.66	95.76 ± 1.66
**Cip**	61.47 ± 19.27	38.53 ± 19.27	127.41 ± 43.28	-27.41 ± 43.28
